# Effect of Platelet-Derived Growth Factor C on Mitochondrial Oxidative Stress Induced by High d-Glucose in Human Aortic Endothelial Cells

**DOI:** 10.3390/ph15050639

**Published:** 2022-05-23

**Authors:** Adriana Grismaldo Rodríguez, Jairo A. Zamudio Rodríguez, Cindy V. Mendieta, Sandra Quijano Gómez, Sandra Sanabria Barrera, Ludis Morales Álvarez

**Affiliations:** 1 Experimental and Computational Biochemistry Group, Faculty of Sciences, Nutrition and Biochemistry Department, Pontificia Universidad Javeriana, Bogotá 110231, Colombia; jzamudior.91@gmail.com (J.A.Z.R.); mendieta-c@javeriana.edu.co (C.V.M.); 2 Department of Clinical Epidemiology and Biostatistics, Pontificia Universidad Javeriana, Bogotá 110231, Colombia; 3 Immunology and Cell Biology Group, Faculty of Sciences, Microbiology Department, Pontificia Universidad Javeriana, Bogotá 110231, Colombia; squijano@javeriana.edu.co; 4 Traslational Biomedical Research Group, Fundación Cardiovascular de Colombia, Floridablanca 680004, Colombia; sandrasanabria@fcv.org

**Keywords:** HAECs, high glucose, PDGF-C, oxidative stress, mitochondrial ROS, Nrf2/Keap1 pathway

## Abstract

Endothelial dysfunction is an early marker for cardiovascular diseases. Hyperglycemia induces endothelial dysfunction, increasing the production of reactive oxygen species. Platelet-derived growth factor C stimulates angiogenesis and revascularization in ischemic tissues of diabetic mice and promotes the migration of progenitors and mature ECs to injury sites; however, the molecular mechanisms of its actions are not described yet. Here, we evaluated the effect of PDGF-C on oxidative stress induced by HG. Human aortic endothelial cells were grown in glucose concentrations ranging from 5 mmol/L to 35 mmol/L for 1 to 24 h. Treatment with 50 ng/mL PDGF-C was done for 1 to 3 h. Cytosolic and mitochondrial ROS were measured by fluorometry, and the expression of antioxidant enzymes was evaluated by Western blot. Nrf2 and Keap1 expression was assessed by real-time PCR. High glucose induced mitochondrial ROS production. PDGF-C diminished the oxidative stress induced by high glucose, increasing SOD2 expression and SOD activity, and modulating the Keap1 expression gene. These results give new evidence about the mitochondrial antioxidant effect that PDGF-C could exert on endothelial cells exposed to high glucose and its considerable role as a therapeutic target in diabetes.

## 1. Introduction

Cardiovascular diseases (CVDs) are considered the leading cause of death worldwide [1,2]. People with diabetes have a two- to-three-fold increase in the risk of developing CVDs compared to healthy people [3]. According to ADA (2021) [4], fasting glucose levels ≥ 126 mg/dL (7 mmol/L), a glucose tolerance test with 75 g of oral glucose ≥ 200 mg/dL (11.1 mM), or aleatory glucose values ≥ 200 mg/dL plus glycosylated hemoglobin > 5.7% are considered criteria for diabetes diagnosis. The condition of acute, transitory, or prolonged hyperglycemia in diabetes is associated with vascular complications, and 75% of these patients will die due to ischemic CVDs, including coronary heart disease, vascular/peripheral arterial disease, and stroke [3,5,6].

A common characteristic of all vascular pathologies is an altered endothelial phenotype known as endothelial dysfunction (ED) [7]. In people with diabetes, ED has been attributed to insulin resistance and increased levels of glucose in plasma or hyperglycemia [8,9]. Hyperglycemia induces an increase in reactive oxygen species (ROS) production, mainly superoxide (O_2_.^-^), not only because of increased activity of the mitochondrial electron transport chain (ETC) but also because of a lower expression and activity of endogenous antioxidants, including superoxide dismutase 2 (SOD2), catalase, and glutathione peroxidase 1 (GPx1), leading to oxidative stress [3]. In this scenario, cellular apoptosis can be triggered [10,11].

Activation of the Nrf2/Keap1 pathway is the primary regulator of antioxidant enzyme expression [12]. The Nrf2/Keap1 pathway is activated by increased ROS production, which leads to Keap1 oxidation and consequently Nrf2 release and translocation from cytoplasm to the nucleus, where it acts as a transcription factor for antioxidant enzymes [13,14,15]. However, the response of Nrf2 under conditions of metabolic stress is weak [16], so molecules that upregulate this pathway may improve endothelial function in diabetes.

Vascular endothelial growth factor (VEGF) is essential for stimulating and maintaining the endothelial functional aspects [17,18,19,20] and mitigating the impact of ROS [21]. However, some studies suggest that signaling mediated by VEGF and its receptor is at least partially inhibited in diabetes [17,18,22]. Therefore, the search for therapeutic strategies that preserve, protect, and improve endothelial functions is relevant to research on hyperglycemia-related CVDs. Recent publications propose PDGF-C as a new growth factor with angiogenic properties—independent of VEGF—that could be of clinical use [23,24].

PDGF is a protein formed by homodimers or heterodimers of A, B, C, and D chains that interacts with three different tyrosine kinase receptors formed by homodimers or heterodimers of α and β polypeptides [25]. PDGF-C acts through PDGFRα and PDGFRαβ receptors [26,27]. Among the positive effects of PDGF-C at the vasculature, stimulation of angiogenesis and revascularization of ischemic tissue have been reported in a diabetic mouse model [28]; stimulation of mature EC migration, promotion of angiogenesis, mobilization of endothelial progenitor cells (EPCs), and increased neovascularization have also been demonstrated in an ischemia model [29]. There are also reports of protecting blood vessels by direct action on different vascular cells in a model of retinal degeneration [30] and the activation of critical signaling pathways in anti-apoptosis and proliferation in endothelial cell (EC) models [31]. However, the molecular mechanisms driven by PDGF-C have not been fully described.

In the present study, a model of endothelium altered by high d-glucose was used to evaluate the role of PDGF-C on cellular mechanisms involved in the mitigation of oxidative stress. Knowledge of the modulatory effect of PDGF-C will allow progress in the molecular mechanisms associated with CVD and the identification of new therapeutic targets aimed at the recovery/mitigation of vascular complications related to pathologies such as diabetes.

## 2. Results

### 2.1. High d-Glucose Induces Mitochondrial ROS Production in HAECs

**Figure 1:**
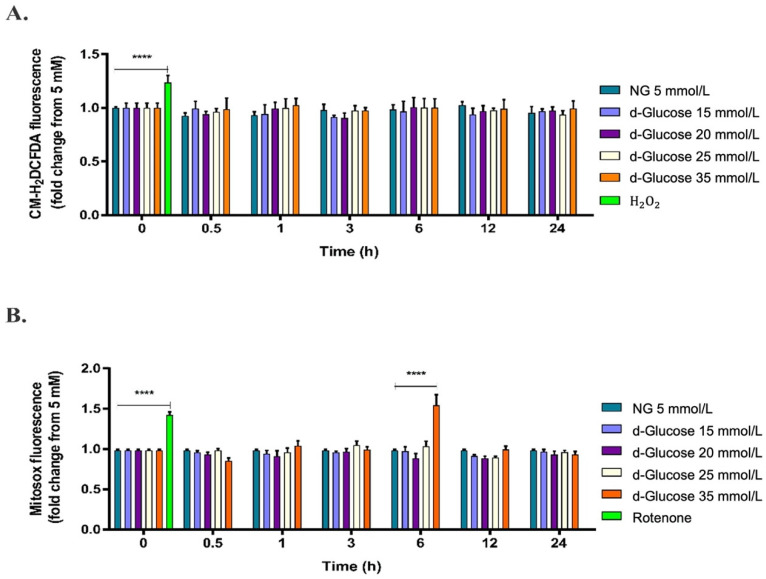
Effect of high d-glucose on cytosolic and mitochondrial ROS production. HAECs were seeded in 96-well plates and exposed to glucose concentrations ranging from 5 mmol/L to 35 mmol/L from 0 to 24 h. (**A**) Cytosolic and (**B**) mitochondrial ROS production was measured by fluorometry with CM-H_2_DCFDA and MitoSOX^TM^, respectively. Treatments with 100 μmol/L H_2_O_2_ or 10 μmol/L rotenone were used as positive controls, respectively. Data are presented as change in mean of fluorescence of each probe regarding 5 mmol/L d-glucose conditions ± SEM of at least three independent experiments (**** *p* < 0.0001).

To evaluate the effect of high d-glucose on cytosolic and mitochondrial ROS production, HAECs were exposed to d-glucose concentrations from 5 mmol/L to 35 mmol/L for 1 to 24 h, and then stained with the probes CM-H_2_DCFDA and MitoSOX. All comparisons were made regarding cells growing in 5 mmol/L glucose, considered a normal fasting blood sugar average [4] (herein referred NG). As shown in Figure 1A, high d-glucose did not affect cytosolic ROS production in HAECs in the times and concentrations evaluated in this study. However, shorter times (less than 1 h), longer times (longer than 24 h), or intervals of the initial screened times (1, 3, 6, 12, and 24 h) were not assayed in this study. Contrarily, as shown in Figure 1B, treatment of cells with 35 mmol/L d-glucose for 6 h significantly increased mitochondrial O_2_^−^ production (**** *p* < 0.0001). According to these results, treatment with 35 mmol/L d-glucose (herein referred to as HG) for 6 h was selected to evaluate the effect of PDGF-C on cellular and mitochondrial events induced by high d-glucose on HAECs. Rotenone, a known complex I of the ETC inhibitor [32], and hydrogen peroxide were used as positive controls for ROS production; effectively, these substances increased the production of O_2_^−^ and H_2_O_2_, respectively (**** *p* < 0.0001). These findings suggest that HG causes oxidative damage by inducing O_2_^−^ production directly from mitochondria in HAECs.

### 2.2. High d-Glucose Increases PDGFRα and PDGFRβ Receptors, but Does Not Induces Changes in PDGF-C in HAECs

**Figure 2:**
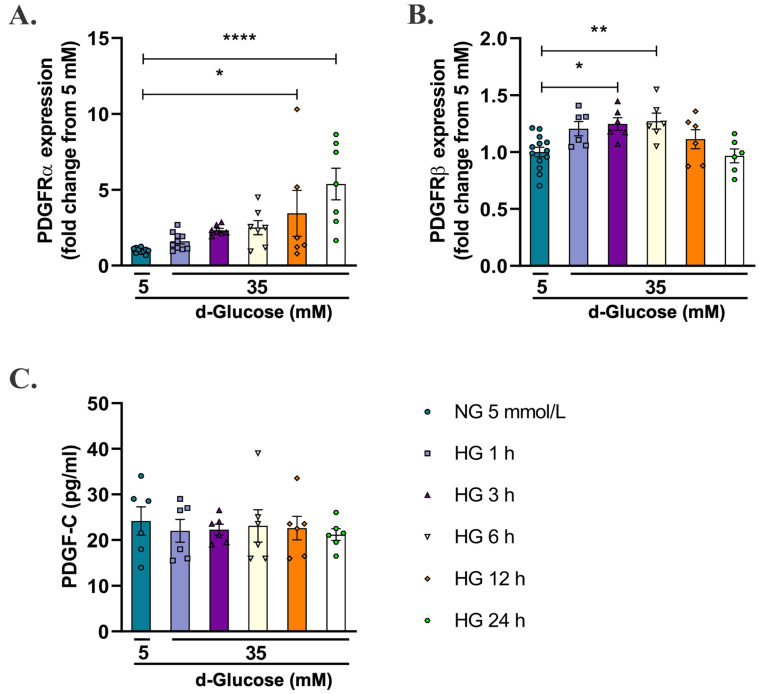
Effect of high d-glucose on endogenous expression of PDGF receptors and content of PDGF-C in the supernatant of endothelial cells. HAECs were seeded in 12-well plates and exposed to 35 mmol/L d-glucose for 1 to 24 h, and expression of PDGFRα (**A**) and PDGFRβ (**B**) was evaluated by flow cytometry. Data are presented as change in mean fluorescence intensity of Alexa Fluor 488 regarding 5 mmol/L d-glucose ± SEM of at least three independent experiments (* *p* < 0.05, ** *p* < 0.001, **** *p* < 0.0001). To measure the content of PDGF-C (**C**), cells were seeded in 96 wells, and supernatants of cells were treated as mentioned before and submitted to ELISA assay. Data are presented as the concentration of PDGF-C in pg/mL and represent the mean ± SEM of at least three independent experiments.

Since 35 mmol/L of d-glucose was the concentration that increased the production of mitochondrial O_2_^−^ in HAECs, the next step was to evaluate its effects on endogenous levels of PDGF receptors (PDGFRα and PDGFRβ) and the ligand PDGF-C. As shown in Figure 2A (Supplementary Figure S1A), HG for 12 h (* *p* < 0.05) and 24 h (**** *p* < 0.0001) increased the levels of PDGFRα regarding NG. Similarly, HG for 3 h (* *p* < 0.05) and 6 h (** *p* < 0.001) increased the levels of PDGFRβ (Figure 2B, Supplementary Figure S1B). On the contrary, no significant changes were observed in the content of PDGF-C ligand in supernatants of HAECs treated with 35 mmol/L of d-glucose in any of the evaluated times compared with cells in NG.

### 2.3. PDGF-C Reduces the Increase of Mitochondrial ROS Production Induced by High d-Glucose

**Figure 3:**
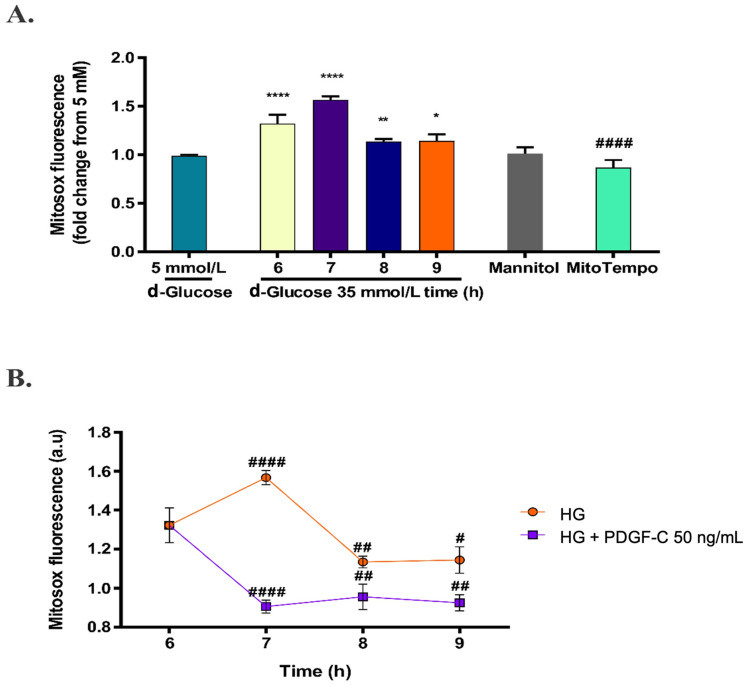
Effect of PDGF-C on mitochondrial ROS production induced by high d-glucose. HAECs were seeded in 96-well plates and exposed to 35 mmol/L d-glucose for 6 to 9 h, without (**A**) and with (**B**) 50 ng/mL PDGF-C. Mitochondrial ROS production was measured each hour by fluorometry with MitoSOX^TM^. A total of 10 μmol/L MitoTEMPO for 1 h before HG treatment and 30 mmol/L mannitol were used as antioxidant and osmolarity controls, respectively. Data with * are presented as change in the mean of fluorescence regarding 5 mmol/L d-glucose ± SEM of at least three independent experiments (**** *p* < 0.0001, ** *p* < 0.001, * *p* < 0.05). Data with # are presented as change in mean of fluorescence regarding 35 mmol/L d-glucose for 6 h ± SEM of at least three independent experiments (#### *p* < 0.0001, ## *p* <0.01, # *p* < 0.05).

Considering the d-glucose conditions determined as mentioned above, the effect of HG on mitochondrial O_2_^−^ production was evaluated in times ranging from 6 to 9 h. As shown in Figure 3A, the increase in mitochondrial superoxide production continued increasing for 7 h (**** *p* < 0.0001), 8 h (** *p* < 0.001), and 9 h (* *p* < 0.05). Cells treated with 30 mmol/L mannitol (added to EBM-2 containing d-glucose 5 mmol/L) for 6 h were used as osmolarity control and compared with HG treatment; there was no effect of osmotic pressure on mitochondrial ROS production. Cells treated with HG for 6 h were pre-treated with mitoTEMPO 10 µmol/L for 1 h and effectively, mitoTEMPO reduced the production of mitochondrial ROS induced by HG for 6 h (#### *p* < 0.0001). Therefore, HAECs exposed to HG for 6 h were treated with 50 ng/mL PDGF-C and ROS production was evaluated each hour for 3 h. PDGF-C significantly diminished mitochondrial superoxide production induced by HG at 7 h (#### *p* < 0.0001), 8 h (## *p* < 0.01), and 9 h (# *p* < 0.05) (Figure 3B). These data suggest that PDGF-C reduces the mitochondrial-generated oxidative stress induced by HG in HAECs.

### 2.4. PDGF-C Up-Regulates SOD2 Antioxidant Enzyme Expression and SOD Activity in HAECs Going on High d-Glucose

**Figure 4:**
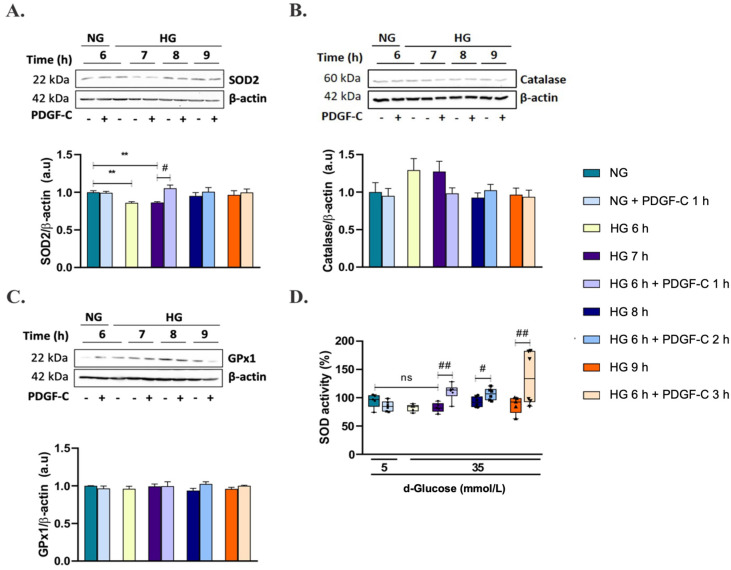
Effect of PDGF-C on antioxidant enzyme expression and SOD activity in endothelial cells treated with high glucose. HAECs were seeded in 6-well plates and exposed to 35 mmol/L d-glucose for 6 to 9 h without and with 50 ng/mL PDGF-C and (**A**) SOD2, (**B**) catalase, and (**C**) GPx1 expression was evaluated by Western blot. Images correspond to representative Western blots for each enzyme. Densitometry analysis corresponds to the band of each enzyme normalized with the band of β-actin for at least 3 independent experiments. Data are presented as the mean ± SEM of at least three independent experiments (** *p* < 0.01 regarding NG, # *p* < 0.05 regarding HG). In the same conditions mentioned above, cells were lysed and (**D**) SOD activity was measured by colorimetric assay. Data are presented as the mean of % of inhibition of formazan formation by SOD ± SEM of at least three independent experiments (## *p* < 0.01, # *p* < 0.05 regarding HG, ns: non-significant).

To determine the mechanisms associated with the reduction of mitochondrial superoxide production by PDGF-C, the expression of the antioxidant enzymes SOD2, catalase, and GPx1 was evaluated by Western blot. As shown in Figure 4A (Supplementary Figure S2), HG for 6 and 7 h significantly reduced SOD2 expression compared to NG (** *p* < 0.01). Treatment with 50 ng/mL PDGF-C for 1 h significantly restored SOD2 expression down-regulated by HG for 6 h (# *p* < 0.05). There was no effect of HG alone or combined with PDGF-C on catalase and GPx1 antioxidant enzyme expression (Figure 4B,C, Supplementary Figures S3 and S4); nevertheless, a non-significantly tendency to increase was observed in catalase when cells were treated with HG for 6 and 7 h (Figure 4B). To investigate whether the role of PDGF-C in reducing HG-induced mitochondrial ROS production was due not only to increased SOD2 enzyme expression but also to its activity, SOD activity was measured as mentioned above. Although HG did not have a significant effect on this parameter, a tendency to reduce its activity was observed at 7 h. Treatment with PDGF-C increased SOD activity in cells treated with HG for 7, 8, and 9 h (## *p* < 0.01, # *p* < 0.05) (Figure 4D). These data suggest that PDGF-C reduces mitochondrial superoxide production induced by HG up-regulating SOD2 expression and increasing SOD activity.

### 2.5. PDGF-C Up-Regulates SOD2 Antioxidant Enzyme Expression and SOD Activity in HAECs Going on High d-Glucose

**Figure 5:**
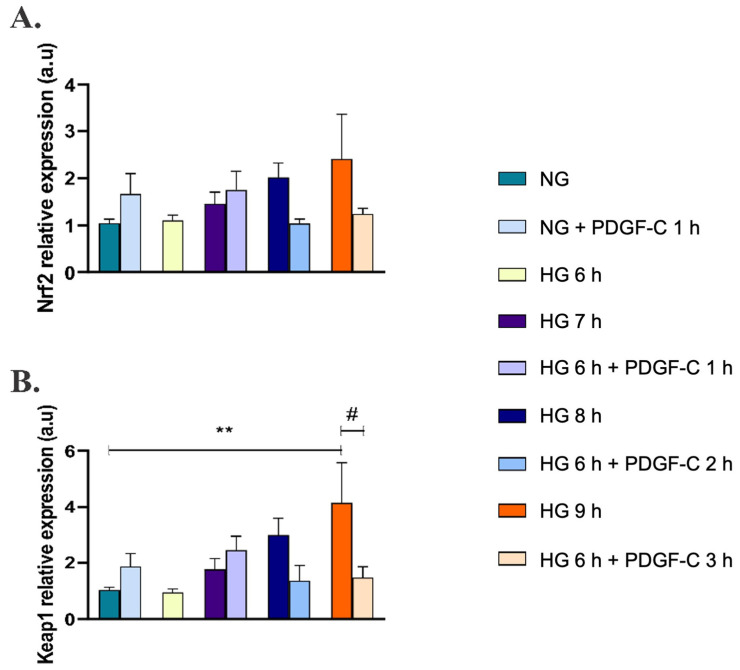
Effect of PDGF-C on Nrf2 and Keap1 gene expression in endothelial cells treated with high glucose. HAECs were seeded in 6-well plates and exposed to 35 mmol/L d-glucose for 6 to 9 h without and with 50 ng/mL PDGF-C. Relative expression of (**A**) Nrf2 and (**B**) Keap1 genes was evaluated by qRT-PCR. Data with ** are presented as a change in expression of each gene regarding 5 mmol/L d-glucose ± SEM of at least three independent experiments (** *p* < 0.01). Data with # are presented as a change in expression of each gene regarding 35 mmol/L d-glucose for 6 h ± SEM of at least three independent experiments (# *p* < 0.05).

To discover the effect of PDGF-C on the Nrf2/Keap1 pathway, the expression of these genes was evaluated by qRT-PCR. As shown in Figure 5A, HG did not induce changes in the relative expression of the Nrf2 gene compared to cells in NG, although a tendency to increase was observed. Likewise, HG for 9 h up-regulated the expression of the Keap1 gene (** *p* < 0.01) (Figure 5B). Interestingly, PDGF-C did not affect Nrf2 expression in any of the assessed conditions (Figure 5A); however, the treatment with this growth factor reduced the high d-glucose overexpression of Keap1 (# *p* < 0.05) (Figure 5B).

## 3. Discussion

One of the risk factors associated with the onset of CVDs in patients with metabolic disorders such as type I and II diabetes is the increase in glucose plasma levels or hyperglycemia [3,6]. ECs constitute the first vascular barrier; thus, the modifications that occur in the intima of the blood vessels are considered the pathological origin of different CVDs [8]. Here, in an in vitro model of HAECs, we confirmed some of the dangerous effects of HG, mainly at the mitochondrial level. Our results show that HG increased mitochondrial ROS production (Figure 1B), which was neutralized by treatment with the SOD2 mimetic MitoTEMPO. Additionally, the expression of genes related to the Nrf2/Keap1 gene pathway was dysregulated, which could be associated with the attenuation of the expression and activity of components of the antioxidant system such as SOD2.

Our study did not replicate the endothelial damage seen with chronic hyperglycemia in diabetic patients. Nevertheless, it is one of the few studies, together with the study of Yano et al. (2004) [33], that simulated the damage induced by acute expositions to high glucose, which frequently occurs in the postprandial state of diabetic patients without treatment, who can reach blood glucose levels higher than 360 mg/dL (20 mmol/L) [34]. These studies show that multiple acute expositions to high glucose concentrations can contribute to oxidative stress and, consequently, to chronic dysfunctional damage observed in the endothelium of people with diabetes.

Although in our model, the increase in glucose concentrations did not induce cytosolic ROS production, likely because of the acute HG treatments evaluated here compared with studies where times of HG exposure were higher than 24 h [35,36,37,38,39,40], it is known that the primary source of ROS in this cellular compartment is the activation of metabolic pathways such as AGE, PKC, hexosamines, and polyoles [41,42]. It is noteworthy that we did not measure cytosolic ROS production in times shorter than 1 h or longer than 24 h either between intervals of the initially screened times (1, 3, 6, 12, and 24 h), which could be considered a limitation. So, our results do not reflect a complete scenario that could demonstrate that HG does not induce increases in this parameter or that the increase is transient. Further studies are needed to explore the contribution of HG to the production of cytosolic ROS in HAECs.

On the other hand, our results show a transient production of mitochondrial ROS in acute exposition to high glucose (6 to 9 h); after 12 h, ROS production diminished to basal levels. This behavior could indicate that cells activate defensive mechanisms to compensate for the damage, including UCP2 protein activation (which normalizes the membrane potential and reduces ROS production) and induction of antioxidant enzyme expression [43]. However, we did not evaluate these mechanisms because our objective was to assess the effect of PDGF-C while high glucose was increasing ROS production.

These observations can also be explained by the unifying mechanisms hypothesis of Brownlee (2005) [44], which states that high glucose-induced damage follows a linear model in which mitochondrial ROS production precedes the cytosolic ROS production [43]. Through different experimental approaches, Brownlee and his lab members demonstrated that inhibition of mitochondrial superoxide production inhibits GAPDH, leading to an accumulation of glycolysis intermediates, which finalizes with the activation of the four major pathways (AGE, PKC, hexosamines, and polyols) and induction of cytosolic ROS production.

Various factors promote the endothelial dysfunction induced by increased production of mitochondrial superoxide, which reacts rapidly with nitric oxide, causing a reduction in its bioavailability because of the formation of peroxynitrite and favoring a predominant state of vasoconstriction [45]. (1) NO is a hydrophobic and highly diffusible gas that can pass easily through biological membranes; thus, it allows autocrine and paracrine effects to be exerted [46]. (2) NO has high affinity for membrane lipids, hemoproteins, thiols, and cysteine-containing proteins found mainly in mitochondria [47]. (3) Although a cytosolic eNOS produces most NO, it has been reported that this enzyme can also be expressed on the outer mitochondrial membrane [48]; this condition generates a closer space for rapid diffusion from the cytosol to mitochondria. (4) An alternative isoform for NOS that generates NO in a calcium-dependent form and is located in the inner mitochondrial membrane has been described previously [47,49].

Therefore, the search for therapeutic strategies that preserve, protect, or improve the characteristics and functions of the endothelium is an exciting area of research in the context of CVD associated with metabolic disorders. The data presented here show for the first time a favorable effect of PDGF-C in reducing HG-induced oxidative stress in HAEC.

PDGF-C is a growth factor that exerts its actions by binding with high affinity to PDGFRα and with lower affinity to PDGFRαβ [26]. Although the expression of PDGFRα in ECs is low compared to other cellular models, such as VSMCs or pericytes [25,28,29,30], it also has been previously reported in limb tissues of diabetic mice [28] and HUVECs in HG conditions [31]. Here we report for the first time the effect of HG on endogenous levels of PDGF receptors and PDGF-C ligand in a model of HAECs (Figure 2). Our results show that acute exposition to HG (<24 h) increases the protein levels of PDGFRα and PDGFRβ. In contrast, there was no HG effect on PDGF-C content in cell supernatants. In this context, Moriya and Ferrara (2015) [31] reported diminished expression of PDGFRα and PDGFRβ in HUVECs exposed to 30 mmol/L of d-glucose for five days, but they did not observe changes in PDGF-C levels regarding NG.

On the other hand, Moriya and Ferrara (2014) [28] did not find differences in PDGFRα expression but showed diminished levels of PDGF-C in a model of diabetic mice regarding control mice. Thus, in our conditions, the increased expression of PDGF receptors and the non-affected basal PDGF-C levels could favor the signal transduction initiated by exogenous PDGF-C and modulate the dangerous biological effects of HG on ECs. In our model, treatment with 50 ng/mL PDGF-C for 1, 2, and 3 h reduced mitochondrial O_2_^−^ production induced by HG to NG levels (Figure 3B). It seemed to be associated with the induction of SOD2 expression, SOD activity (Figure 4A,D), and down-regulation of the Keap1 gene (Figure 5B). These results suggest an important role of PDGF-C and its signaling in the cardiovascular damage induced by high glucose.

SOD is a family of enzymes that catalyzes the formation of H_2_O_2_ and O_2_ from O_2_^−^ and H_2_O_2_ [50]. In NG conditions, it has been reported that growth factors such as VEGF induce the expression of SOD2 through a mechanism coupled to Nf-κβ in human coronary artery endothelial cells (HCAECs) and HUVECs, suggesting an essential role of VEGF signaling in the redox status of ECs [21]. However, VEGF and its receptor signaling are disrupted in diabetes due to the excess of ROS induced by HG, which results in the phosphorylation of vascular endothelial growth factor receptor 2 (VEGFR2) at the intracellular level, reducing its availability at the cell surface [17].

There are few reports about the effect of PDGF on the reduction of oxidative stress. In this context, Cabezas et al. (2017) [32], in a model of rotenone-induced ROS in human astrocytes, showed the effect of PDGF-B in the decrease of nucleic acid oxidation measured by 8-OHdG. Additionally, in this model, the attenuation of the peroxynitrite-mediated protein modifications was induced by rotenone and it was reported that this effect was mediated by the induction of antioxidant enzyme expression. Our results report for the first time a PDGF-C effect on the pro-oxidant HG-induced status on ECs. At this point, PDGF-C could be an attractive alternative for reducing the risk of the development of CVDs, such as coronary disease, peripheral arterial disease, and stroke, associated with the increase of ROS in diabetes.

Antioxidant protein expression is regulated mainly by the Nrf2/Keap1 pathway [13,14,15], which could be initiated by the activation of PDGFRα [13]. In our conditions, HG seemed to induce the expression of the Nrf2 gene (Figure 5A), which could have been related to a compensatory mechanism and a cell-specific antioxidant response [16,51]; similarly, HG increased the Keap1 gene expression at 9 h (Figure 5B), which is known for retaining Nrf2 in the cytoplasm [52]. Importantly, PDGF-C down-regulated the expression of Keap1 in the cells’ ongoing HG (Figure 5B), which could have facilitated the translocation of Nrf2 to the nucleus and the transcription of antioxidant enzymes such as SOD2. Nevertheless, further analyses using a Keap1-specific inhibitor, such as CPUY192018 [53], are necessary to clearly stablish the role of PDGF-C on the activation of the Nrf2/Keap1 pathway in our model.

Our results support the potential antioxidant role that PDGF-C could exert in repairing the damage induced by high glucose in vascular complications associated with metabolic pathologies like diabetes.

## 4. Materials and Methods

### 4.1. Cells and Reagents

Human aortic endothelial cells (CC-2535), EGM-2 BulletKit (CC-3162) and EBM-2 (00190860) were obtained from Lonza (Walkersville, MD, USA). hrPDGF-C (SRP3139), Rotenone (R8875), and MitoTEMPO (SML0737) were obtained from Sigma-Aldrich (St. Louis, MO, USA). CM-H_2_DCFDA 5-(and-6)-chloromethyl-2′,7′-dichlorodihydrofluorescein diacetate, acetyl ester (C6827), MitoSOX^TM^ (M36008), anti-rabbit IgG Alexa Fluor 488 (A11008), SuperSignal^TM^ West Pico PLUS chemiluminescent substrate (34577), TRIzol^TM^ reagent (15596-026), and M-MLV reverse transcriptase (28025013) were obtained from ThermoFisher Scientific/Invitrogen (Chelmsford, MA, USA). Oligo (dt)18 primer (38029) and DNTPs mix (39029) were obtained from Meridian Life Sciences (Memphis, TN, USA). FastStar essential DNA Green Master (06402712001) was obtained from Applied Biosystems (Waltham, CA, USA). Quantikine ELISA human PDGF-CC (DC00) was obtained from R & D Systems (Minneapolis, MN, USA). A Superoxide Dismutase Activity Assay Kit was obtained from Abcam (ab65354) (Cambridge, UK). Antibodies against PDGFRα (D1E1E), PDGFRβ (28E1), SOD2 (D3X8F XP^®^), catalase (D4P7B XP^®^), GPx1, β actin (8H10D10), Anti-rabbit IgG HRP-linked, and anti-mouse IgG HRP-linked were obtained from Cell Signaling Technology (Danvers, MA, USA).

### 4.2. Cell Culture and Treatments

Human aortic endothelial cells (HAECs) at passage 3 were cultured in EGM-2 BulletKit containing 5.5 mmol/L glucose, 2% fetal bovine serum (FBS), hydrocortisone, hFGF-B, VEGF, R3-IGF-1, ascorbic acid, hEGF, heparin, and gentamicin/amphotericin and incubated at 37 °C in humidified 5% CO_2_ in the air. The medium was changed every 3 to 4 days. Once cultures reached 80% confluence, cells were trypsinized and used to establish the assays. Cells at a maximum of 6 passages were used for all experiments. Cells were seeded in multi-well plates and, after 24 h, the culture medium was changed for EBM-2 containing 5.5 mmol/L glucose and 0.2% fetal bovine serum. After 12 h, cells were treated with different d-glucose concentrations, ranging from 5.5 mmol/L to 35 mmol/L in EBM-2, from 1 h to 24 h. As an osmotic control, mannitol 30 mmol/L added to EBM-2 containing d-glucose 5 mmol/L was used for some experiments. After stress induction with 35 mmol/L of d-glucose for 6 h, treatments with 50 ng/mL of PDGF-C were assessed for 1, 2, and 3 h, considered the short half-life of PDGF in HUVECs, which has been reported to be between 50 min and 3 h [54]. All comparisons were made from cells treated with 5.5 mmol/L of glucose considered the control group.

### 4.3. Reactive Oxygen Species (ROS) Production

HAECs were seeded in 96-well plates in the culture conditions mentioned above. Cytosolic and mitochondrial ROS production were evaluated with CM-H_2_DCFDA and MitoSOX^TM^ dyes, respectively, accordingly to provider instructions. Briefly, cells were washed once with PBS and incubated with 10 μmol/L CM-H_2_DCFDA for 30 min or 4 μmol/L MitoSOX^TM^ for 20 min. Fluorescence emission was measured in a FLUOstar Omega microplate reader at 535 nm for CM-H_2_DCFDA and 510 nm for MitoSOX, using 100 μmol/L H_2_O_2_ or 10 μmol/L rotenone for 1 h as positive controls for ROS production. Cells treated with MitoTEMPO 10 μmol/L for 1 h before d-glucose treatment were used as negative control only for mitochondrial ROS production. Fluorescence emission was normalized to protein concentration per well measured by Bradford assay.

### 4.4. PDGF α and β Receptor Expressions by Flow Cytometry

Protein expression of PDGFRα and PDGFRβ was measured by flow cytometry. HAECs were seeded in 12-well plates and treated as mentioned above. Cells were fixed with 1.6% formaldehyde for 20 min and permeabilized with absolute methanol at −20 °C for 1 h. Cells were washed twice with 2% FBS in PBS and incubated at 4 °C overnight with PDGFRα (dil 1:100) and PDGFRβ (dil 1:100) antibodies. Anti-rabbit IgG Alexa Fluor 488 (dil 1:500) was used as a secondary antibody and incubated at room temperature for 1 h. After washing once, cells were resuspended in PBS and acquired in a GuavaEasyCyte (Millipore, MA, USA) flow cytometer. Mean fluorescence intensity was analyzed for each treatment with InCyte software.

### 4.5. Determination of PDGF-C in Cell Supernatants

The content of PDGF-C in supernatants of HAECs was measured by Quantikine ELISA Human PDGF-CC. Briefly, 50 µL of supernatants of cells growing in 96-well plates and treated as mentioned above were transferred to a microplate pre-coated with a monoclonal antibody specific for PDGF-C. After several washes, an enzyme-link monoclonal antibody for PDGF-C was added to each well, which reacted with a substrate solution and produced a blue color in proportion to the amount of PDGF-C bound in the initial step. Absorbance at 450 nm was measured in a FLUOstar Omega microplate reader.

### 4.6. Antioxidant Protein Expression by Western Blot

Protein expression of antioxidant enzymes SOD2, catalase, and GPx1 was measured by Western blot. HAECs were seeded in 6-well plates and treated as mentioned above. After a wash with cold PBS, cells were scraped with RIPA buffer on ice. After sonication and centrifugation at 4 °C, cell lysate was collected, and protein was quantified by bicinchoninic acid. Equivalent protein quantities were subjected to SDS-polyacrylamide gels and transferred to PVDF membranes. Membranes were incubated at 4 °C overnight with the following antibodies: SOD2 (dil 1:1000), catalase (dil 1:1000), and GPx1 (dil 1:1000). β-actin (1:2000) was used as the loading control. Anti-rabbit IgG HRP-linked (1:5000) and anti-mouse IgG HRP-linked (1:5000) were used as secondary antibodies and incubated at room temperature for 1 h. Signals were revealed with SuperSignal^TM^ West Pico PLUS chemiluminescent substrate and captured by the iBright 1500 imaging system from ThermoFisher Scientific (Chelmsford, MA, USA). Western blot densitometry was measured by Image J [55].

### 4.7. SOD Activity

SOD activity was measured by a Superoxide Dismutase Activity Assay Kit (colorimetric) in a FLUOstar Omega microplate reader, using total lysates of HAECs treated as mentioned above and according to manufacturer instructions. This assay uses the activity of xanthine oxidase to produce the superoxide anion that is catalyzed by SOD to produce hydrogen peroxide and molecular oxygen. Superoxide anion also acts on a tetrazolium salt to produce a formazan dye that is detected by absorbance at 450 nm. The greater the activity of SOD in the sample, the less formazan dye is produced.

### 4.8. Gene Expression Assay

Relative expression of Nrf2 and Keap1 genes was evaluated by qRT-PCR. Quantification and purity of RNA obtained by TRIzol isolation were evaluated in a NanoDrop 2000 (ThermoFisher Scientific, Waltham, MA, USA). cDNA was synthesized from 400 ng of RNA for each treatment according to M-MLV reverse transcriptase instructions. qRT-PCR was carried out using the FastStar essential DNA Green Master in a CFX96 Touch Real-Time System (Biorad, Hercules, CA, USA) following this protocol: denaturation at 95 °C for 10 min, 45 cycles at 95 °C for 10 s, 60 °C for 10 s, and 72 °C for 10 s. The relative expression of each gene was normalized with Rpl27 gene expression using the 2^−∆∆Ct^ comparative method [56]. Primer sequences used for these assays are listed in Table 1.

### 4.9. Statistics

All experiments were performed at least in triplicate and data are expressed as mean ± SEM. One-way ANOVA was used for between-group comparisons, followed by Bonferroni’s post hoc test. An unpaired t-test was used for comparisons between two groups. A value of *p* < 0.05 was considered statistically significant. Graph Pad Software (San Diego, CA) was used for all analyses.

## 5. Conclusions

**Figure 6:**
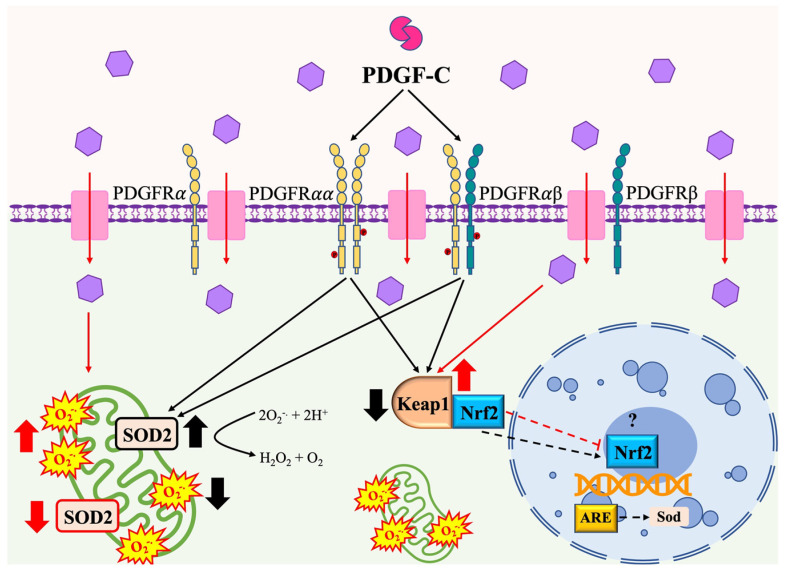
PDGF-C effects on high d-glucose-induced oxidative stress in HAECS. Read description in the text.

The main findings of our study are shown in Figure 6. In summary, high d-glucose produces oxidative stress in HAECs that is observed as an increase in superoxide anion produced by mitochondria and a reduction in SOD2 protein expression, SOD activity, and up-regulated Keap1 gene expression (red arrows). PDGF-C treatment reduces HG-induced mitochondrial ROS production, restoring SOD2 expression and increasing its activity. It is relevant to highlight that the effect of PDGF-C was associated with the reduction of Keap1 overexpression induced by a high content of d-glucose (black arrows), which could allow the translocation of Nrf2 from the cytoplasm to the nucleus, where it acts as a transcription factor for antioxidant enzymes. It remains unknown whether PDGF-C-mediated increased SOD2 expression is associated with Nrf2 translocation (dashed arrows).

## Figures and Tables

**Table 1:** Primer sequences for expression of Nrf2 and Keap1 genes.

Gen	Forward Sequence	Reverse Sequence
Nrf2	TCCAGTCAGAAACCAGTGGAT	GAATGTCTGCGCCAAAAGCTG
Keap1	CAACTTCGCTGAGCAGATTGGC	TGATGAGGGTCACCAGTTGGCA
Rpl27	ATCGCCAAGAGATCAAAGATAA	TCTGAAGACATCCTTATTGACG

## Data Availability

Data is contained within the article and supplementary material.

## Supplementary Materials

The following supporting information can be downloaded at: https://www.mdpi.com/article/10.3390/ph15050639/s1, Figure S1: Representative fluorescence histograms for evaluation of (**A**) PDGFRα and (**B**) PDGFRβ expression. Figure S2: Original triplicate blots for evaluation of SOD2 expression. Figure S3: Original triplicate blots for evaluation of Catalase expression. Figure S4: Original triplicate blots for evaluation of GPx1 expression.

## References

[1] Virani S.S., Alonso A., Aparicio H.J., Benjamin E.J., Bittencourt M.S., Callaway C.W., Carson A.P., Chamberlain A.M., Cheng S., Delling F.N. (2021). Heart Disease and Stroke Statistics—2021 Update: A Report from the American Heart Association. Circulation.

[2] GBD 2019 Diseases and Injuries Collaborators (2020). Global burden of 369 diseases and injuries in 204 countries and territories, 1990–2019: A systematic analysis for the Global Burden of Disease Study 2019. Lancet.

[3] Dal Canto E., Ceriello A., Rydén L., Ferrini M., Hansen T.B., Schnell O., Standl E., Beulens J.W. (2019). Diabetes as a cardiovascular risk factor: An overview of global trends of macro and micro vascular complications. Eur. J. Prev. Cardiol..

[4] American Diabetes Association (2021). 2. Classification and Diagnosis of Diabetes: Standards of Medical Care in Diabetes—2021. Diabetes Care.

[5] Giglio R.V., Stoian A.P., Haluzik M., Pafili K., Patti A.M., Rizvi A.A., Ciaccio M., Papanas N., Rizzo M. (2021). Novel molecular markers of cardiovascular disease risk in type 2 diabetes mellitus. Biochim. Biophys. Acta Mol. Basis Dis..

[6] Galicia-Garcia U., Benito-Vicente A., Jebari S., Larrea-Sebal A., Siddiqi H., Uribe K.B., Ostolaza H., Martín C. (2020). Pathophysiology of Type 2 Diabetes Mellitus. Int. J. Mol. Sci..

[7] Tang X., Luo Y.X., Chen H.-Z., Liu D.-P. (2014). Mitochondria, endothelial cell function, and vascular diseases. Front. Physiol..

[8] Domingueti C.P., Dusse L.M.S., Carvalho M.D.G., Sousa L.P., Gomes K.B., Fernandes A.P. (2016). Diabetes mellitus: The linkage between oxidative stress, inflammation, hypercoagulability and vascular complications. J. Diabetes Complicat..

[9] Patel H., Chen J., Das K.C., Kavdia M. (2013). Hyperglycemia induces differential change in oxidative stress at gene expression and functional levels in HUVEC and HMVEC. Cardiovasc. Diabetol..

[10] He L., He T., Farrar S., Ji L., Liu T., Ma X. (2017). Antioxidants Maintain Cellular Redox Homeostasis by Elimination of Reactive Oxygen Species. Cell. Physiol. Biochem..

[11] Avogaro A., Albiero M., Menegazzo L., de Kreutzenberg S., Fadini G.P. (2011). Endothelial Dysfunction in Diabetes: The role of reparatory mechanisms. Diabetes Care.

[12] Vashi R., Patel B.M. (2021). NRF2 in Cardiovascular Diseases: A Ray of Hope!. J. Cardiovasc. Transl. Res..

[13] Haider N., Larose L. (2020). Activation of the PDGFRα-Nrf2 pathway mediates impaired adipocyte differentiation in bone marrow mesenchymal stem cells lacking Nck1. Cell Commun. Signal..

[14] Sharma A., Rizky L., Stefanovic N., Tate M., Ritchie R.H., Ward K.W., de Haan J.B. (2017). The nuclear factor (erythroid-derived 2)-like 2 (Nrf2) activator dh404 protects against diabetes-induced endothelial dysfunction. Cardiovasc. Diabetol..

[15] Kovac S., Angelova P.R., Holmström K.M., Zhang Y., Dinkova-Kostova A.T., Abramov A.Y. (2015). Nrf2 regulates ROS production by mitochondria and NADPH oxidase. Biochim. Biophys. Acta.

[16] Ungvari Z., Bailey-Downs L., Gautam T., Jimenez R., Losonczy G., Zhang C., Ballabh P., Recchia F.A., Wilkerson D.C., Sonntag W.E. (2011). Adaptive induction of NF-E2-related factor-2-driven antioxidant genes in endothelial cells in response to hyperglycemia. Am. J. Physiol. Heart Circ. Physiol..

[17] Warren C.M., Ziyad S., Briot A., Der A., Iruela-Arispe M.L. (2014). A Ligand-Independent VEGFR2 Signaling Pathway Limits Angiogenic Responses in Diabetes. Sci. Signal..

[18] Moriya J., Ferrara N. (2014). Inhibiting the Response to VEGF in Diabetes. Sci. Signal..

[19] Eichmann A., Simons M. (2012). VEGF signaling inside vascular endothelial cells and beyond. Curr. Opin. Cell Biol..

[20] Ferrara N., Gerber H.-P., LeCouter J. (2003). The biology of VEGF and its receptors. Nat. Med..

[21] Abid M.R., Schoots I.G., Spokes K.C., Wu S.-Q., Mawhinney C., Aird W.C. (2004). Vascular Endothelial Growth Factor-mediated Induction of Manganese Superoxide Dismutase Occurs through Redox-dependent Regulation of Forkhead and IκB/NF-κB. J. Biol. Chem..

[22] Waltenberger J. (2009). VEGF resistance as a molecular basis to explain the angiogenesis paradox in diabetes mellitus. Biochem. Soc. Trans..

[23] Li X., Kumar A., Zhang F., Lee C., Li Y., Tang Z., Arjunan P. (2010). VEGF-independent angiogenic pathways induced by PDGF-C. Oncotarget.

[24] Dimmeler S. (2005). Platelet-Derived Growth Factor CC—A Clinically Useful Angiogenic Factor at Last?. N. Engl. J. Med..

[25] Andrae J., Gallini R., Betsholtz C. (2008). Role of platelet-derived growth factors in physiology and medicine. Genes Dev..

[26] Folestad E., Kunath A., Wågsäter D. (2018). PDGF-C and PDGF-D signaling in vascular diseases and animal models. Mol. Asp. Med..

[27] Lee C., Zhang F., Tang Z., Liu Y., Li X. (2013). PDGF-C: A new performer in the neurovascular interplay. Trends Mol. Med..

[28] Moriya J., Wu X., Zavala-Solorio J., Ross J., Liang X.H., Ferrara N. (2014). Platelet-derived growth factor C promotes revascularization in ischemic limbs of diabetic mice. J. Vasc. Surg..

[29] Li X., Tjwa M., Moons L., Fons P., Noel A., Ny A., Zhou J.M., Lennartsson J., Li H., Luttun A. (2005). Revascularization of ischemic tissues by PDGF-CC via effects on endothelial cells and their progenitors. J. Clin. Investig..

[30] He C., Zhao C., Kumar A., Lee C., Chen M., Huang L., Wang J., Ren X., Jiang Y., Chen W. (2014). Vasoprotective effect of PDGF-CC mediated by HMOX1 rescues retinal degeneration. Proc. Natl. Acad. Sci. USA.

[31] Moriya J., Ferrara N. (2015). Inhibition of protein kinase C enhances angiogenesis induced by platelet-derived growth factor C in hyperglycemic endothelial cells. Cardiovasc. Diabetol..

[32] Cabezas R., Vega-Vela N.E., González-Sanmiguel J., González J., Esquinas P., Echeverria V., Barreto G.E. (2018). PDGF-BB Preserves Mitochondrial Morphology, Attenuates ROS Production, and Upregulates Neuroglobin in an Astrocytic Model Under Rotenone Insult. Mol. Neurobiol..

[33] Yano M., Hasegawa G., Ishii M., Yamasaki M., Fukui M., Nakamura N., Yoshikawa T. (2004). Short-term exposure of high glucose concentration induces generation of reactive oxygen species in endothelial cells: Implication for the oxidative stress associated with postprandial hyperglycemia. Redox Rep..

[34] Choi S.-W., Benzie I.F., Ma S.-W., Strain J.J., Hannigan B.M. (2008). Acute hyperglycemia and oxidative stress: Direct cause and effect?. Free Radic. Biol. Med..

[35] Chen F., Ma D., Li A. (2020). SENP3 regulates high glucose-induced endothelial dysfunction via ROS dependent signaling. Diabetes Vasc. Dis. Res..

[36] Zhang M., Jin X., Zhang Z., Li B., Yang G. (2018). Vildagliptin protects endothelial cells against high glucose-induced damage. Biomed. Pharmacother..

[37] Ke S.-Y., Yu S.-J., Liu D.-H., Shi G.-Y., Wang M., Zhou B., Wu L., Song Z.-M., Zhu J.-M., Wu C.-D. (2021). Ginsenoside Rb1 Protects Human Umbilical Vein Endothelial Cells against High Glucose-Induced Mitochondria-Related Apoptosis through Activating SIRT3 Signalling Pathway. Chin. J. Integr. Med..

[38] Lin F., Yang Y., Wei S., Huang X., Peng Z., Ke X., Zeng Z., Song Y. (2020). Hydrogen Sulfide Protects Against High Glucose-Induced Human Umbilical Vein Endothelial Cell Injury Through Activating PI3K/Akt/eNOS Pathway. Drug Des. Dev. Ther..

[39] Liu D., Wu M., Lu Y., Xian T., Wang Y., Huang B., Zeng G., Huang Q. (2017). Protective effects of 6-Gingerol on vascular endothelial cell injury induced by high glucose via activation of PI3K-AKT-eNOS pathway in human umbilical vein endothelial cells. Biomed. Pharmacother..

[40] Qin W., Ren B., Wang S., Liang S., He B., Shi X., Wang L., Liang J., Wu F. (2016). Apigenin and naringenin ameliorate PKCβII-associated endothelial dysfunction via regulating ROS/caspase-3 and NO pathway in endothelial cells exposed to high glucose. Vasc. Pharmacol..

[41] Triggle C.R., Ding H., Marei I., Anderson T.J., Hollenberg M.D. (2020). Why the endothelium? The endothelium as a target to reduce diabetes-associated vascular disease. Can. J. Physiol. Pharmacol..

[42] Liu H., Xiang H., Zhao S., Sang H., Lv F., Chen R., Shu Z., Chen A.F., Chen S., Lu H. (2019). Vildagliptin improves high glucose-induced endothelial mitochondrial dysfunction via inhibiting mitochondrial fission. J. Cell. Mol. Med..

[43] Gero D., Lenasi H. (2017). Hyperglycemia-Induced Endothelial Dysfunction. Endothelial Dysfunction-Old Concepts and New Challenges.

[44] Brownlee M. (2005). The Pathobiology of Diabetic Complications. Diabetes.

[45] Cahill P.A., Redmond E.M. (2016). Vascular endothelium—Gatekeeper of vessel health. Atherosclerosis.

[46] Brookes P.S. (2004). Mitochondrial nitric oxide synthase. Mitochondrion.

[47] Ghafourifar P., Cadenas E. (2005). Mitochondrial nitric oxide synthase. Trends Pharmacol. Sci..

[48] Gao S., Chen J., Brodsky S.V., Huang H., Adler S., Lee J.H., Dhadwal N., Cohen-Gould L., Gross S.S., Goligorsky M.S. (2004). Docking of Endothelial Nitric Oxide Synthase (eNOS) to the Mitochondrial Outer Membrane: A pentabasic amino acid sequence in the autoinhibitory domain of eNOS targets a proteinase K-cleavable peptide on the cytoplasmic face of mitochondria. J. Biol. Chem..

[49] Haynes V., Elfering S.L., Squires R.J., Traaseth N., Solien J., Ettl A., Giulivi C. (2003). Mitochondrial Nitric-oxide Synthase: Role in Pathophysiology. IUBMB Life.

[50] Wang Y., Branicky R., Noe A., Hekimi S. (2018). Superoxide dismutases: Dual roles in controlling ROS damage and regulating ROS signaling. J. Cell Biol..

[51] Satta S., Mahmoud A.M., Wilkinson F.L., Yvonne Alexander M., White S.J. (2017). The role of Nrf2 in cardiovascular function and disease. Oxid. Med. Cell. Longev..

[52] Chen B., Lu Y., Chen Y., Cheng J. (2015). The role of Nrf2 in oxidative stress-induced endothelial injuries. J. Endocrinol..

[53] Hui Q., Karlstetter M., Xu Z., Yang J., Zhou L., Eilken H.M., Terjung C., Cho H., Gong J., Lai M.J. (2020). Inhibition of the Keap1-Nrf2 protein-protein interaction protects retinal cells and ameliorates retinal ischemia-reperfusion injury. Free Radic. Biol. Med..

[54] Gay C.G., Winkles J.A. (1991). The half-lives of platelet-derived growth factor A- and B-chain mRNAS are similar in endothelial cells and unaffected by heparin-binding growth factor-1 or cycloheximide. J. Cell. Physiol..

[55] Schneider C.A., Rasband W.S., Eliceiri K.W. (2012). NIH Image to ImageJ: 25 Years of image analysis. Nat. Methods.

[56] Livak K.J., Schmittgen T.D. (2001). Analysis of relative gene expression data using real-time quantitative PCR and the 2^−ΔΔCT^ Method. Methods.

