# Supplementary material for: Effect of Platelet-Derived Growth Factor C on Mitochondrial Oxidative Stress Induced by High d-Glucose in Human Aortic Endothelial Cells
Source: Pharmaceuticals (Basel). 2022 May 23;15(5):639. doi: 10.3390/ph15050639 (PMC9143891; doi:10.3390/ph15050639)
Supplement: Supplementary file 1 [file pharmaceuticals-15-00639-s001.zip › pharmaceuticals-1689509-supplementary.pdf]

Supplementary Material

Effect of Platelet-Derived Growth Factor C on Mitochondrial Oxidative Stress Induced by High d-Glucose in Human Aortic Endothelial Cells

Adriana Grismaldo Rodríguez [1,*], Jairo A. Zamudio Rodríguez [1], Cindy V. Mendieta [1,2], Sandra Quijano Gómez [3], Sandra Sanabria Barrera [4] and Ludis Morales Álvarez [1,*]

[1] Experimental and Computational Biochemistry Group, Faculty of Sciences, Nutrition and Biochemistry Department, Pontificia Universidad Javeriana, Bogotá 110231, Colombia; jzamudior.91@gmail.com (J.A.Z.R.); mendieta-c@javeriana.edu.co (C.V.M.)
[2] Department of Clinical Epidemiology and Biostatistics, Pontificia Universidad Javeriana, Bogotá 110231, Colombia
[3] Immunology and Cell Biology Group, Faculty of Sciences, Microbiology Department, Pontificia Universidad Javeriana, Bogotá 110231, Colombia; squijano@javeriana.edu.co
[4] Traslational Biomedical Research Group, Fundación Cardiovascular de Colombia, Santander 681001 Colombia; sandrasanabria@fcv.org
* Correspondence: mgrismaldo@javeriana.edu.co (A.G.R.); ludis.morales@javeriana.edu.co (L.M.Á.); Tel.: +57-3114566976 (A.G.R.); +57-3132107272 (L.M.Á.)

Figure S1. Representative fluorescence histograms for evaluation of (**A**) PDGFRα and (**B**) PDGFRβ expression

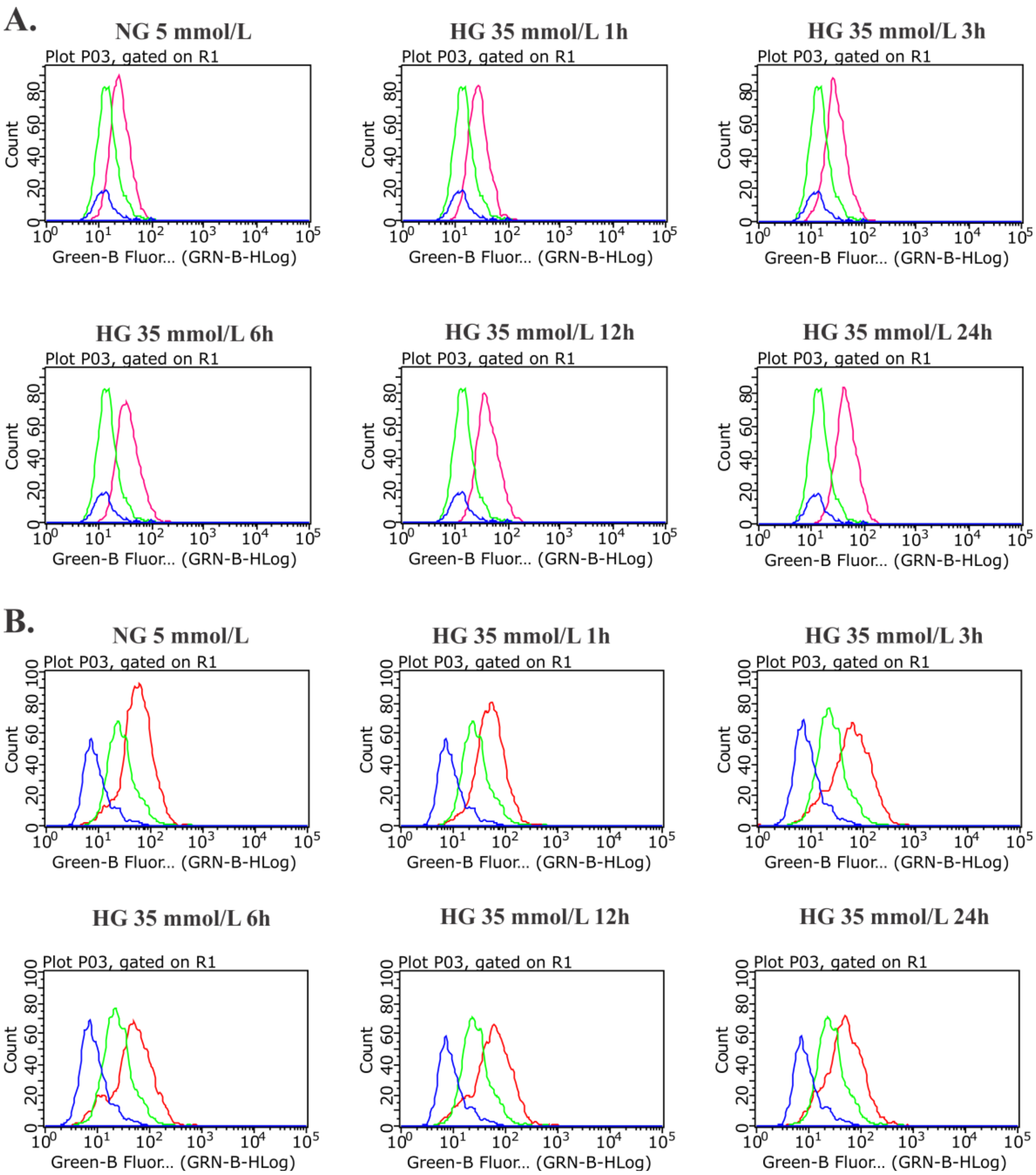

The blue histograms represent cells without staining, the green histograms represent cells incubated with Alexa Fluor 488 secondary antibody and the red histograms represents (A) PDGFRα and (B) PDGFRβ expression (cells incubated with primary antibody plus secondary antibody).

Figure S2. Original triplicate blots for evaluation of SOD2 expression

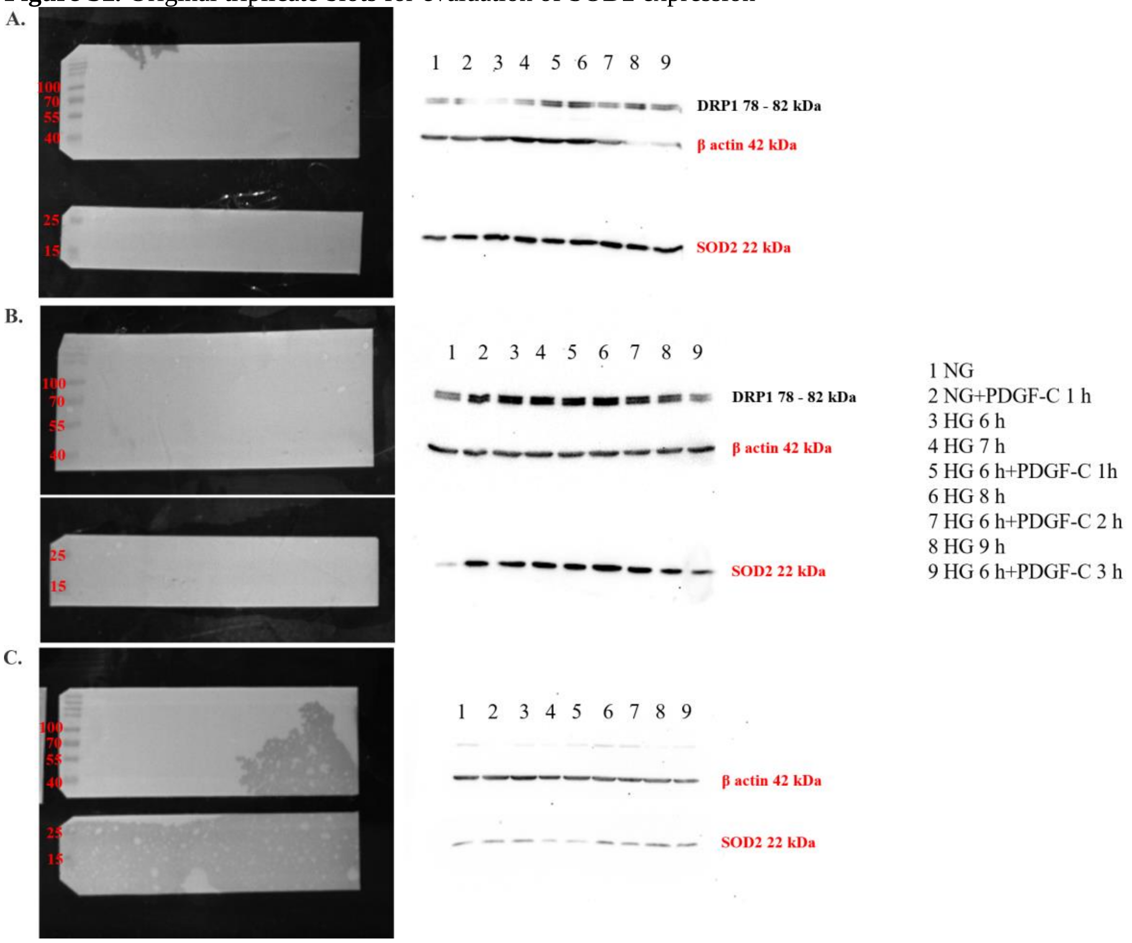

As shown in Figure 4A, blot C was selected as the representative blot for the manuscript

Figure S3. Original triplicate blots for evaluation of Catalase expression

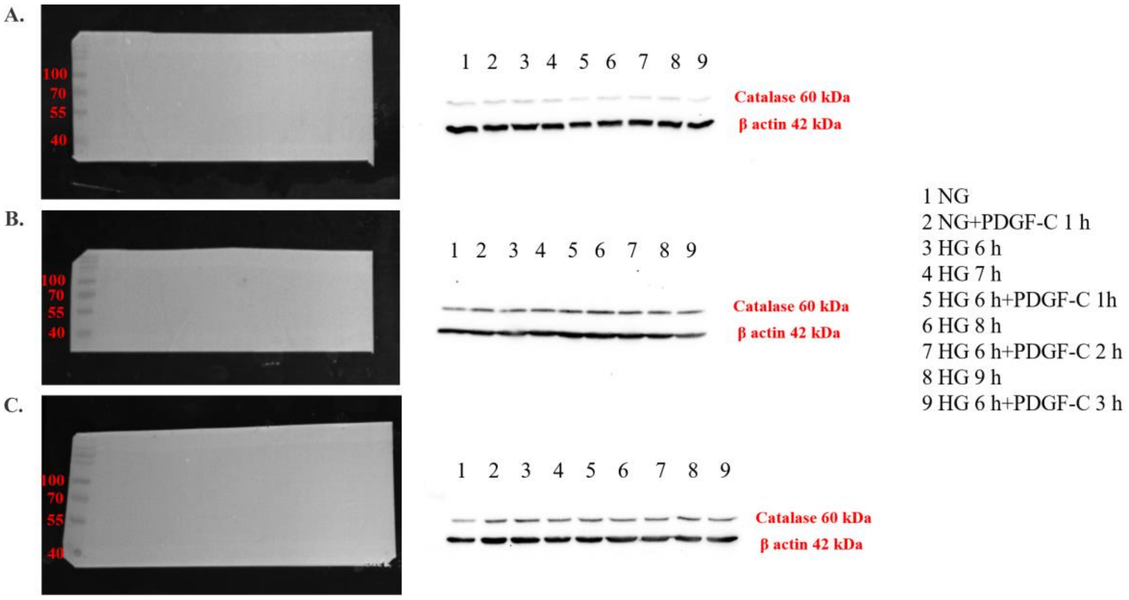

As shown in Figure 4B, blot A was selected as the representative blot for the manuscript

Figure S4. Original triplicate blots for evaluation of GPx1 expression

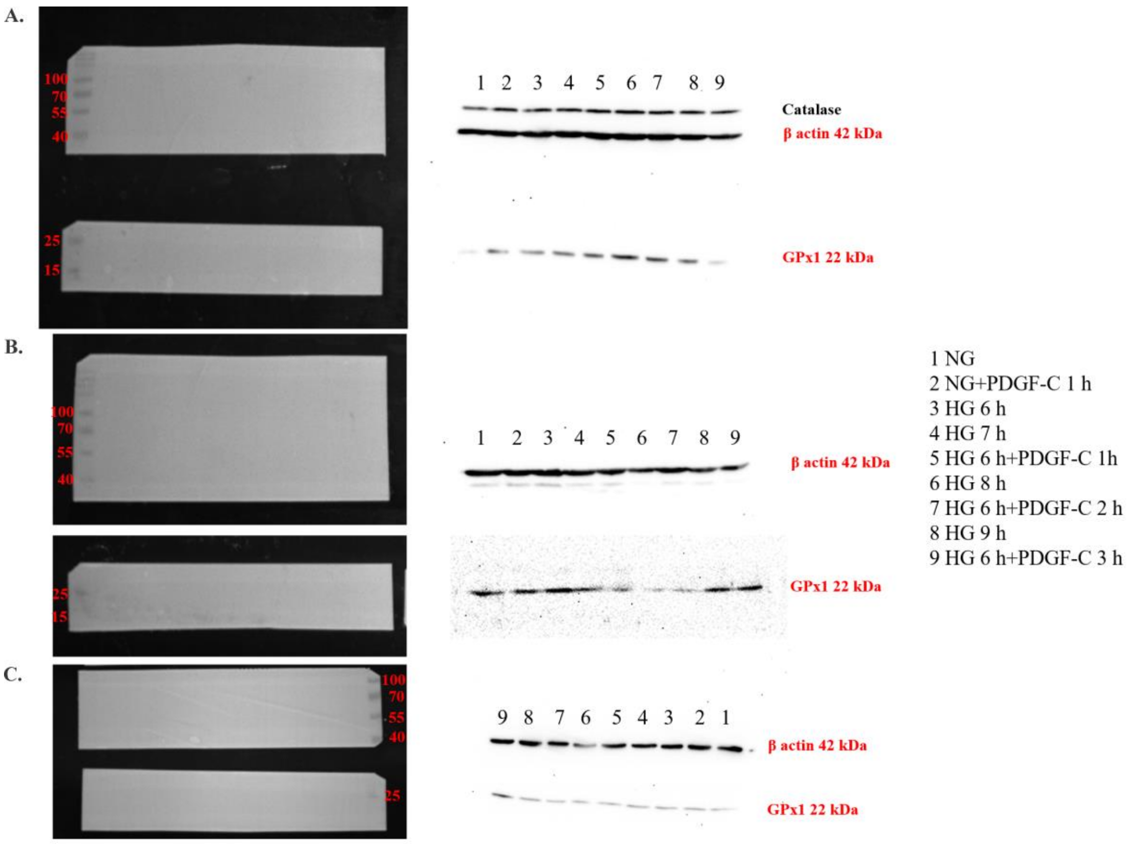

As shown in Figure 4C, blot A was selected as the representative blot for the manuscript. Note that blot C is on the contrary sense.
